# Platycodin D confers oxaliplatin Resistance in Colorectal Cancer by activating the LATS2/YAP1 axis of the hippo signaling pathway

**DOI:** 10.7150/jca.77322

**Published:** 2023-01-22

**Authors:** Chien-Hao Wang, Rathinasamy Baskaran, Shawn Shang-Chuan Ng, Tso-Fu Wang, Chi-Cheng Li, Tsung-Jung Ho, Dennis Jine-Yuan Hsieh, Chia-Hua Kuo, Ming-Cheng Chen, Chih-Yang Huang

**Affiliations:** 1 Department of Chinese Medicine, Hualien Tzu Chi Hospital, Buddhist Tzu Chi Medical Foundation, Tzu Chi University, Hualien, Taiwan; 2 Integration Center of Traditional Chinese and Modern Medicine, Hualien Tzu Chi Hospital, Buddhist Tzu Chi Medical Foundation, Hualien, Taiwan; 3 Department of Bioinformatics and Medical Engineering, Asia University, Taichung, Taiwan; 4 Department of Biological Science and Technology, College of Life Sciences, China Medical University, Taichung, Taiwan; 5 Ph.D. Program for Biotechnology Industry, China Medical University, Taichung 406, Taiwan; 6 Department of Hematology and Oncology, Hualien Tzu Chi Hospital, Buddhist Tzu Chi Medical Foundation, Hualien, Taiwan; 7 School of Medicine Tzu Chi University, 701, Section 3, Chung-Yang Road, Hualien 97004, Taiwan; 8 Center of Stem Cell & Precision Medicine, Hualien Tzu Chi Hospital, Buddhist Tzu Chi Medical Foundation, Hualien, Taiwan; 9 Chinese Medicine, Hualien Tzu Chi Hospital, Buddhist Tzu Chi Medical Foundation, Tzu Chi University, Hualien, Taiwan; 10 Department of Medical Laboratory Science and Biotechnology, China Medical University, Taichung, Taiwan; 11 School of Medical Laboratory and Biotechnology, Chung Shan Medical University, Taichung, Taiwan; 12 Clinical Laboratory, Chung Shan Medical University Hospital, Taichung 402, Taiwan; 13 Laboratory of Exercise Biochemistry, University of Taipei, Taipei, Taiwan; 14 Department of Kinesiology and Health Science, College of William and Mary, Williamsburg, VA, USA; 15 Department of Surgery, Division of Colorectal Surgery, Taichung Veterans General Hospital, Taichung, Taiwan; 16 Faculty of Medicine, National Yang-Ming University, Taipei, Taiwan.; 17 Cardiovascular and Mitochondria related diseases research center, Hualien Tzu Chi Hospital, Hualien 970, Taiwan; 18 Graduate Institute of Biomedicine, China Medical University, Taichung, Taiwan; 19 Department of Biotechnology, Asia University, Taichung 413, Taiwan; 20 Center of General Education, Buddhist Tzu Chi Medical Foundation, Tzu Chi University of Science and Technology, Hualien 970, Taiwan; 21 Department of Medical Research, China Medical University Hospital, China Medical University, Taichung 404, Taiwan

**Keywords:** Oxaliplatin, Drug resistance, Platycodin, Cell cycle, metastasis

## Abstract

Oxaliplatin-based therapy is used as a first-line drug to treat metastatic colorectal cancer. However, long-term and repeated drug treatment resulted in drug resistance and the failure of chemotherapy. Various natural compounds were previously reported to act as chemosensitizers to reverse drug resistance. In this study, we found that platycodin D (PD), a saponin found in *Platycodon grandiflorum*, inhibited LoVo and OR-LoVo cells proliferation, invasion, and migration ability. Our results indicated that combined treatment of oxaliplatin with PD dramatically reduced the cellular proliferation in both LoVo and OR-LoVo cells. Furthermore, treatment with PD dose-dependently decreased LATS2/YAP1 hippo signaling and survival marker p-AKT expression, as well as increased cyclin-dependent kinase inhibitor proteins such as p21 and p27 expression. Importantly, PD activates and promotes YAP1 degradation through the ubiquitination and proteasome pathway. The nuclear transactivation of YAP was significantly reduced under PD treatment, leading to transcriptional inhibition of the downstream genes regulating cell proliferation, pro-survival, and metastasis. In conclusion, our results showed that PD is suitable as a promising agent for overcoming oxaliplatin-resistant colorectal cancer.

## Introduction

Colorectal cancer (CRC) is the third prevalent malignant tumor, causing malignancies. In spite of substantial advancements in CRC therapy, it remains one of the primary causes of cancer-related mortality 1. Oxaliplatin-based chemotherapy is employed in the front lines of CRC treatment all over the world. Nearly half of CRC patients receiving oxaliplatin-based chemotherapy are cured of CRC 2, 3. Oxaliplatin is a platinum based third generation chemotherapeutic agent, which acts against the cancer cells by interacting with DNA and forms cross-links between the two strands in the S phase of cell division 4. On the other hand, chemotherapy could also develop resistance to oxaliplatin, which may allow cancer cells to survive or quiescence, contributing to the cancer reappearance. Resistance to oxaliplatin is likely related to cellular transport, detoxification, DNA repair, cell death, and epigenetic alternation 5, 6. Understanding these molecular mechanisms of oxaliplatin resistance and utilizing them for developing novel therapeutic strategies for cancer therapies have been investigated 7-9.

The key serine/threonine kinase of the hippo tumor-suppressive signaling pathway is Large tumor suppressor 2 (LATS2), which is present in chromosome 13Q1.11 in humans. LATS2 controls the cell cycle by regulating Yes-associated protein 1 (YAP) and Transcriptional coactivator with PDZ-binding motif (TAZ) (orthologues of Yorkie in Drosophila) phosphorylation, which are key downstream regulators in the hippo signaling pathway 10. LATS2 is a key regulator of mitotic progression and activates its downstream proteins such as YAP, retinoblastoma protein (pRB), and p53, which altogether contribute to cell cycle arrest and cancer cell growth inhibition 11. Further, LATS2 is also known to interact with other signaling pathways like estrogen signaling, and the Ras and Akt network which plays role in regulating cell proliferation, apoptosis, and metastasis of different cancer types 12, 13. Recent studies have shown the association of hippo pathway in the development of CRC 14-16.

The remarkable oncogenic characteristics of the Hippo signaling pathway proteins, such YAP and TAZ, as well as their druggability, are gaining attention in recent researches in cancer drug resistance 17. Paclitaxel and cisplatin resistance is conferred by overexpressing YAP-S127A in ovarian cancer cells with low baseline YAP activity, but YAP knockdown in ovarian cancer cells with higher YAP activity improves sensitivity to paclitaxel and cisplatin. This is due to YAP-S127A lacks a significant LATS1/2 phosphorylation site and accumulates in the nucleus 18. In nasopharyngeal cancer, epithelial-mesenchymal transition (EMT) and overexpression of TAZ have been found to be positively correlated 19. While TAZ stimulates interleukin-8 (IL-8) transcription to develop resistance to doxorubicin, YAP induces doxorubicin resistance by triggering the mitogen-activated protein kinase (MAPK) pathway 20.

Natural products have been extensively studied in the realm of drug discovery because they are a rich source of molecules with a wide structural variety. *In vitro* and *in vivo* anticancer properties have been observed in a wide range of natural compounds 21-23. *Platycodon grandifloras* common Chinese medicinal plant belongs to the family Campanulaceae which was used in traditional folk medicines in China, Japan, and Korea. The root of *Platycodon grandifloras* is used to cure a variety of ailments heavy cough, sore throat, bronchitis, and asthma 24, 25. Platycodin D is one of the major saponins presented in *Platycodon grandifloras* which possess various pharmacological properties such as anti-oxidant, anti-inflammatory, anti-obesity, anti-atherogenic, and immunomodulatory effects 26-30. PD has remarkable antitumor effects on several cancer cell lines, reducing the proliferation of cancer cell growth by inhibiting the cell cycle and inducing apoptosis 31, 32.

On the basis of these studies, we hypothesize that LoVo colorectal cancer cell line develops resistance to chemotherapeutic drugs by activating the hippo signaling pathway and activates LATS2, and YAP by phosphorylation and PD treatment in the parental and resistance LoVo cells could effectively modulate the hippo signaling pathway by inhibiting the phosphorylation of LATS2, and YAP.

## Materials and Methods

### Cell culture

Food Industry Research and Development Institute, Hsinchu, Taiwan, provided the LoVo colon cell line, which is human colon cell line. LoVo cells were cultured in Dulbecco's Minimum Essential Medium (DMEM, Sigma, USA) containing 10% fetal bovine serum (FBS) (HyClone^TM^, USA), streptomycin (100 g/mL) and penicillin (100 U/mL) at 37 °C in a humidified environment of 5% CO_2_. To establish stable colon cancer cell lines resistant to oxaliplatin, LoVo cell lines were exposed to oxaliplatin in dose-dependent manner from 0 to 25 µM for 24 hr. This was MTT-1. LoVo cell lines exposed to 21.5 µM oxaliplatin resulted around 50% cell death (IC_50_ of oxaliplatin). Cells treated with 21.5 µM oxaliplatin (24 hr) allowed to reach 80% confluence and passaged twice in this same concentration (21.5 µM) of oxaliplatin. The same procedure was repeated at increased doses of oxaliplatin (30 and 40 µM) until a cell population was selected that demonstrated at least a threefold greater IC_50_ (75 µM) to oxaliplatin than the parental cell lines. OXA-R LoVo cells were developed by the previous report 3.

### MTT assay

To assess the cell viability of LoVo cells, the MTT [3-(4, 5-Dimethylthiazol-2-yl)-2, 5-diphenyltetrazolium-bromide] assay was performed. A 96-well plate was seeded with 1 x 10^4^ parental LoVo cells and 1 x 10^4^ OXA-R LoVo cells, and the cells were exposed to various drug doses. Oxaliplatin was treated to parental LoVo and OXA-R LoVo cells at 1, 5, 10, 15, 20, 25, 30, 40, 50, 60, 70, and 80 µM for 24 hours, After the treatment time, DMEM was removed from the cells and washed with PBS. Each well received 20 μl (5 mg/mL) of MTT, which was then left to incubate for 4 hours. A microplate reader was used to detect the absorbance at 570 nm after dissolving MTT formazan crystals in 200 μl of DMSO. Platycodin D (purity ≥98%, Sigma, USA) was dissolved in DMSO.

### Western blot

An equal amount (30-40 µg) of protein was separated by using an 8-12% SDS-PAGE electrophoresis gel at 90V for 45 minutes. Protein from the gel was transferred to the polyvinylidene fluoride membrane at 4 °C using blotting apparatus (Bio-Rad Laboratories, Hercules, CA, USA). PVDF membrane was then submerged in 5% non-fat milk powder in TBST at room temperature for 1 hr. The membrane was then washed thrice with TBS 3 times, for 5 minutes each. The membrane was then incubated overnight with primary antibody (1:1000 dilution in TBST) at 4 °C in a mechanical rocker. Then, the membrane was washed thrice with TBS 3 times, for 5 minutes each, and incubated with HRP-conjugated secondary antibody (1:5000 dilution in TBST) at room temperature for 1 h in a mechanical rocker. After washing with TBS, the membrane was submerged in chemiluminescence ECL solution (Merck Millipore, Burlington, MA, USA), the protein bands were visualized using chemi-doc apparatus (Fuji-film LAS-3000, GE Healthcare), and densitometric analysis was performed using ImageJ software (version 1.4.3.67) (NIH, Bethesda, MD, USA). Antibodies details: LATS2 (#5888, Cell Signaling, Baltimore, MD, USA), p-LATS2 (ab111344, Abcam, Cambridge, UK), YAP (sc-101199, Santa Cruz Biotechnology, Santa Cruz, CA, USA), p-YAP (#13008, Cell Signaling, Baltimore, MD, USA), TAZ (sc-48805, Santa Cruz Biotechnology, Santa Cruz, CA, USA), p21 (sc-6246, Santa Cruz Biotechnology, Santa Cruz, CA, USA), p27 (sc-1641, Santa Cruz Biotechnology, Santa Cruz, CA, USA), p-AKT (#9275s, Cell Signaling, Baltimore, MD, USA), Ki67 (ab15580, Abcam, Cambridge, UK), β-Actin (sc-47778, Santa Cruz Biotechnology, Santa Cruz, CA, USA), HDAC1 (sc-6298, Santa Cruz Biotechnology, Santa Cruz, CA, USA), Ubiquitin (sc-8017, Santa Cruz Biotechnology, Santa Cruz, CA, USA), GAPDH (sc-25778, Santa Cruz Biotechnology, Santa Cruz, CA, USA).

### Terminal Deoxynucleotidyl Transferase-mediated Nick-End Labeling (TUNEL) assay

DNA breaks due to apoptosis was determined using Terminal Deoxynucleotidyl Transferase-mediated Nick-End Labeling (TUNEL) assay. *In Situ* Cell Death Detection Kit (Roche) was used to assay. After the cells were treated with appropriate dose and time DMEM was removed from the cells and washed with PBS thrice. Then the cells were fixed in 4% formalin for 1 hr. After fixation the cells were made permeable with permeation solution (0.1% Triton X‐100 in 0.1% sodium citrate) for 10 mins. Cells were washed with PBS and incubated in TUNEL solution for 1 hr at room temperature. Cells were stained with nuclear stain DAPI for 5 mins and observed under fluorescent microscope for TUNEL positive cells.

### Transwell migration and invasion assays

Serum-free medium was applied to dilute the Matrigel, and 50 μl of diluted Matrigel was inoculated into each chamber. After being treated with DMSO and PD, both parental LoVo and OXA-R LoVo cells were resuspended at a density of 1 × 105 cells/mL with DMEM medium without FBS. Then, we added 0.2 mL of the cell suspension to each upper chamber of the 24-well plate, while 0.6 mL DMEM containing 20% FBS was added to the lower chambers. After 24 hr, the upper chambers were washed, fixed with 4% paraformaldehyde for 20 min, stained with 0.25% crystal violet for 30 min, and imaged by a microscope.

### Immunofluorescence microscopy

1 × 10^5^ parental or OXA‐resistant LoVo colon cancer cells were seeded 24 well plates and treated with PD for 24 hrs. At the end of treatment cells were washed with PBS and fixed with 4% paraformaldehyde for 1 hr in room temperature. After PBS was cells were permeabilized with permeabilization solution for 10 mins. To prevent non-specific binding, cells were incubated with 10% FBS for 1 hr at room temperature. 250 μl of YAP primary antibody (1:200 dilution) was added to the cells and incubated at 37°C for 3 hrs. Cells were then washed with PBS thrice and incubated with 300 μl FITC conjugated secondary antibody (1:1000 dilution) for 1 hr at room temperature. Cells were then washed and stained with DAPI for 5 mins and washed again with PBS thrice. Cells were then visualized under fluorescent microscope.

### Statistical analysis

The results shown are the means ±SD of three independent experiments. Statistical analysis was performed by one-way analysis of variants followed by a Tukey's post-hoc test SPSS 16 software (SPSS, Chicago, IL, USA).

## Results

### Characterization of chemoresistance in OXP-LoVo colorectal cancer cells

**Figure 1:**
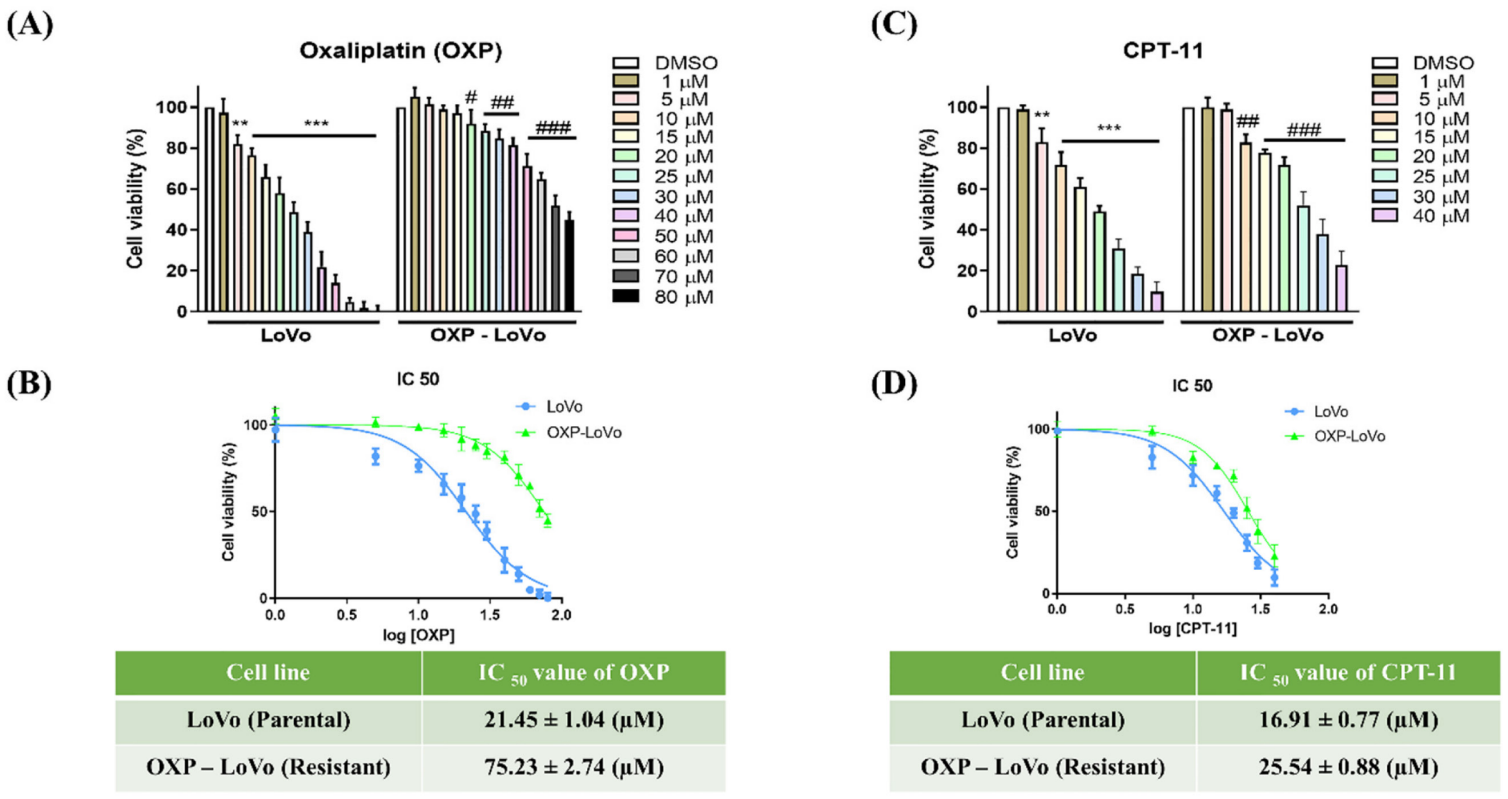
Characterization of chemoresistance in OXP-LoVo colon-rectal cancer cells. (A&C) Parental and OXP-LoVo cells were treated with various concentrations of oxaliplatin (1-80 μM) and CPT-11 (1-40 μM) for 24 hrs. and cell viability was determined by MTT assay. (B&D) IC_50_ values of oxaliplatin (OXP) and CPT-11 in LoVo and OXP-LoVo colon-rectal cancer cells. *** P<0.001, and ** P<0.01 compared to LoVo control group and ### P<0.001, ## P<0.01 and # P<0.05 compared to OXP-LoVo control.

OXP-LoVo colorectal cancer cells were developed based on our previous study 3. Both parental and OXP-LoVo cells were treated with various concentrations of oxaliplatin (1-80 μM) for 24 hrs. and cell viability was determined by MTT assay. Oxaliplatin from 5 μM induces significant cell death in LoVo cells, however in OXP-LoVo cells cell significant cell death was observed after 20 μM of oxaliplatin (Figure 1A). IC_50_ values of oxaliplatin in LoVo and OXP-LoVo was found to be 21.45 μM and 75.23 μM respectively (Figure 1B). In order to establish multidrug resistance, parental LoVo and OXP-LoVo cells were treated with different concentrations of irinotecan (CPT‐11) (1-40 μM) for 24 hrs and cell viability was quantified. 5 μM of CPT-11 induces cell death significantly in parental LoVo cells but in OXP-LoVo cells 10 μM of CPT-11 induced significant cell death. IC_50_ of CPT-11 parental LoVo was found to be 16.91 μM, whereas in OXP-LoVo cells it was around 25.5 μM (Figure 1C&D). This result demonstrated that OXP-LoVo cells were resistant to both OXA and CPT-11.

### PD alone and combined with oxaliplatin inhibits CRC cell proliferation in parental and OXA-resistance cells

**Figure 2:**
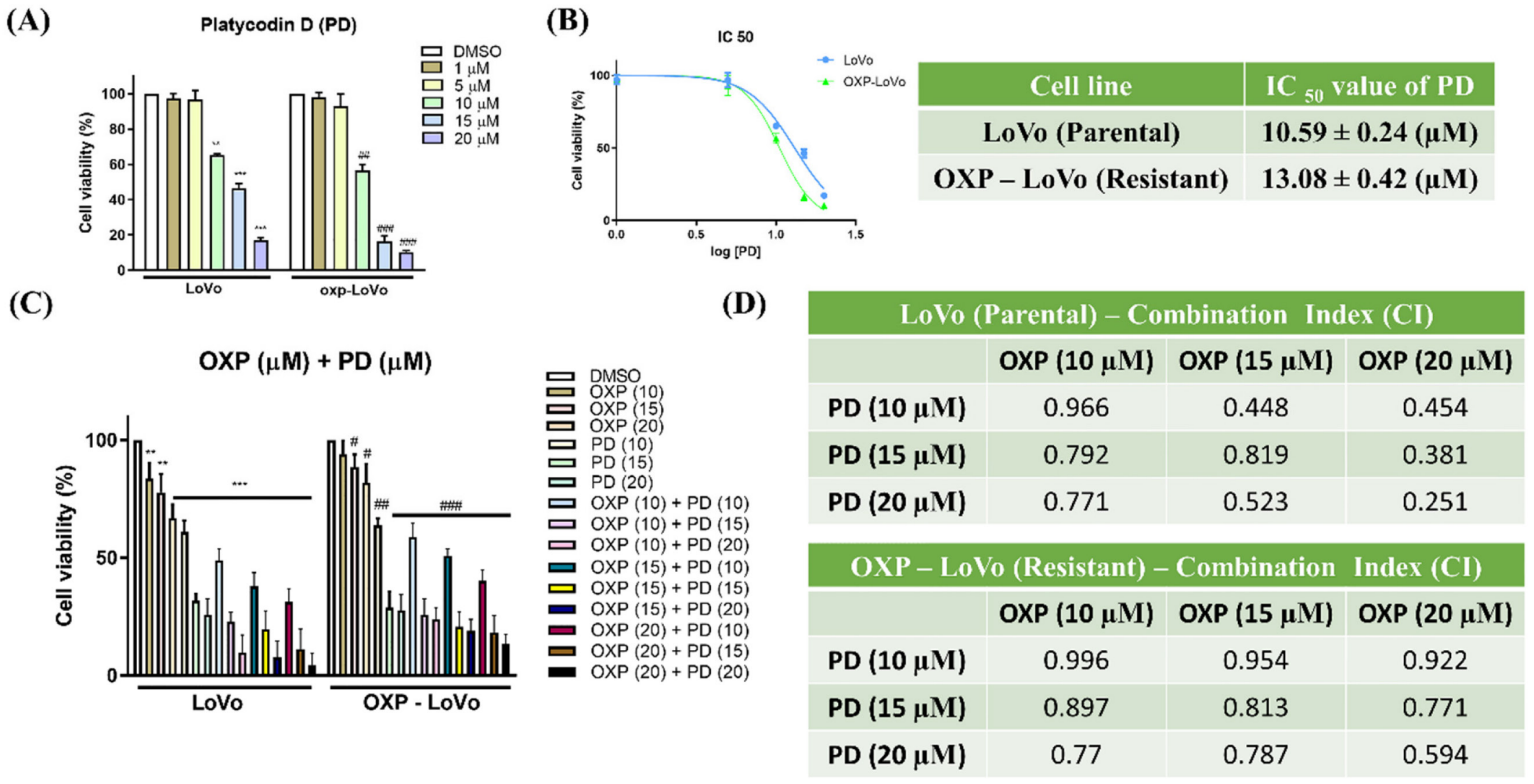
PD alone and combined with oxaliplatin inhibits CRC cell proliferation in parental and OXA-resistance cells. (A) Both parental LoVo and OXP-LoVo cells were treated with different concentrations of PD (1-20 μM) for 24 hrs. (B) IC_50_ of PD in parental LoVo and OXP-LoVo cells. (C) Combinatorial synergistic effect of OXA (10, 15 & 20 μM) and PD (10, 15 & 20 μM) on LoVo and OXP-LoVo cells. (D) A combination index (CI) was calculated for the drug pair OXA and PD. *** P<0.001, and ** P<0.01 compared to LoVo control group and ### P<0.001, and ## P<0.01 compared to OXP-LoVo control.

Several reports have established the anticancer effect of PD 31. In the present study, we evaluated the effect of PD on parental LoVo and OXP-LoVo cell viability. Both parental LoVo and OXP-LoVo cells were treated with different concentrations of PD (1-20 μM) for 24 hrs. In both cells, PD from 10 μM induces cell death significantly and the IC_50_ of PD in parental LoVo and OXP-LoVo cells was found to be 10.59 μM and 13.08 μM respectively (Figure 2A&B). In order to find the combinatorial synergistic effect both the cells were treated combined with OXA (10, 15 & 20 μM) and PD (10, 15 & 20 μM) for 24 hrs, and cell viability was estimated (Figure 2C). Combined treatment of OXA and PD induces cell death in a dose-dependent manner. A combination index (CI) was calculated for the drug pair OXA and PD. CI < 1 is believed to be better compatibility with high synergistic effects. In our present study, CI was found to be below 1 for OXA (10, 15 & 20 μM) and PD (10, 15 & 20 μM) in parental LoVo and OXP-LoVo cells (Figure 2D).

### LATS2 and YAP/TAZ hippo signaling is highly activated in OXP-LoVo cells

**Figure 3:**
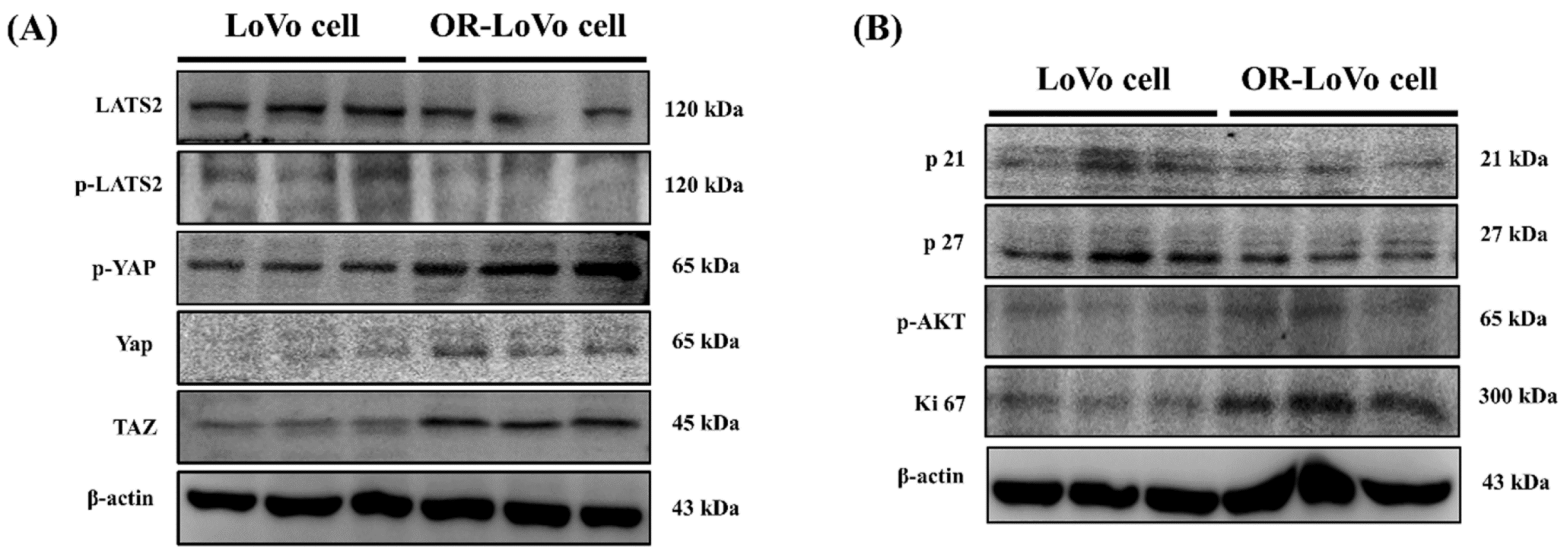
LATS2 and YAP/TAZ hippo signaling is highly activated in OXP-LoVo cells. (A) Immunoblot showing the expression levels of p-LAST2, p-YAP, and TAZ. (B) Immunoblot showing the expression levels of cell cycle and survival proteins p21, p27, p-AKT and Ki67.

We evaluated the hippo signaling pathway proteins and cell cycle regulating protein expression in LoVo and OXP-LoVo cells. p-LAST2 p-YAP and TAZ were upregulated in OXP-LoVo cells when compared to LoVo cells (Figure 3A). On the other hand, cy-clin-dependent kinase inhibitor proteins such as p21 and p27 and cell survival protein p-Akt and Ki 67 were increased in OXP-LoVo cells than parental LoVo cells (Figure 3B).

### PD significantly downregulated LATS2 and YAP/TAZ hippo signaling in LoVo and OXP-LoVo cells

**Figure 4:**
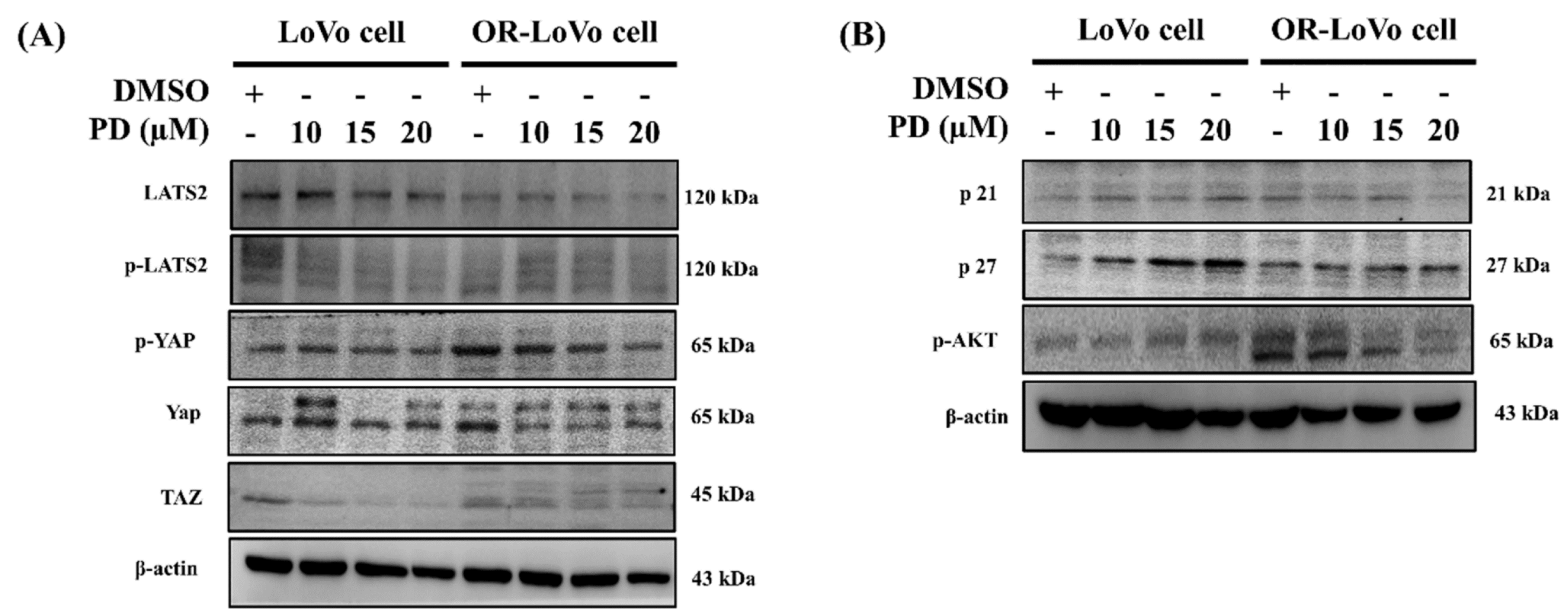
PD significantly downregulated LATS2 and YAP/TAZ hippo signaling in LoVo and OXP-LoVo cells. Western blot analysis showing expression of p-LAST2, p-YAP, and TAZ proteins. (B) Western blot analysis for the protein expressions p21, p27, and p-AKT.

Then we evaluated the effect of PD on hippo signaling, cell cycle, and cell survival protein expression. PD dose-dependently decreased LATS2/YAP1 and Taz in hippo signaling and survival marker p-AKT expression, as well as increased cyclin-dependent kinase inhibitor proteins such as p21 and p27 expression in parental and OXP-LoVo cells (Figure 4).

### PD induces apoptosis, invasion, and migration in LoVo and OXP-LoVo cells

**Figure 5:**
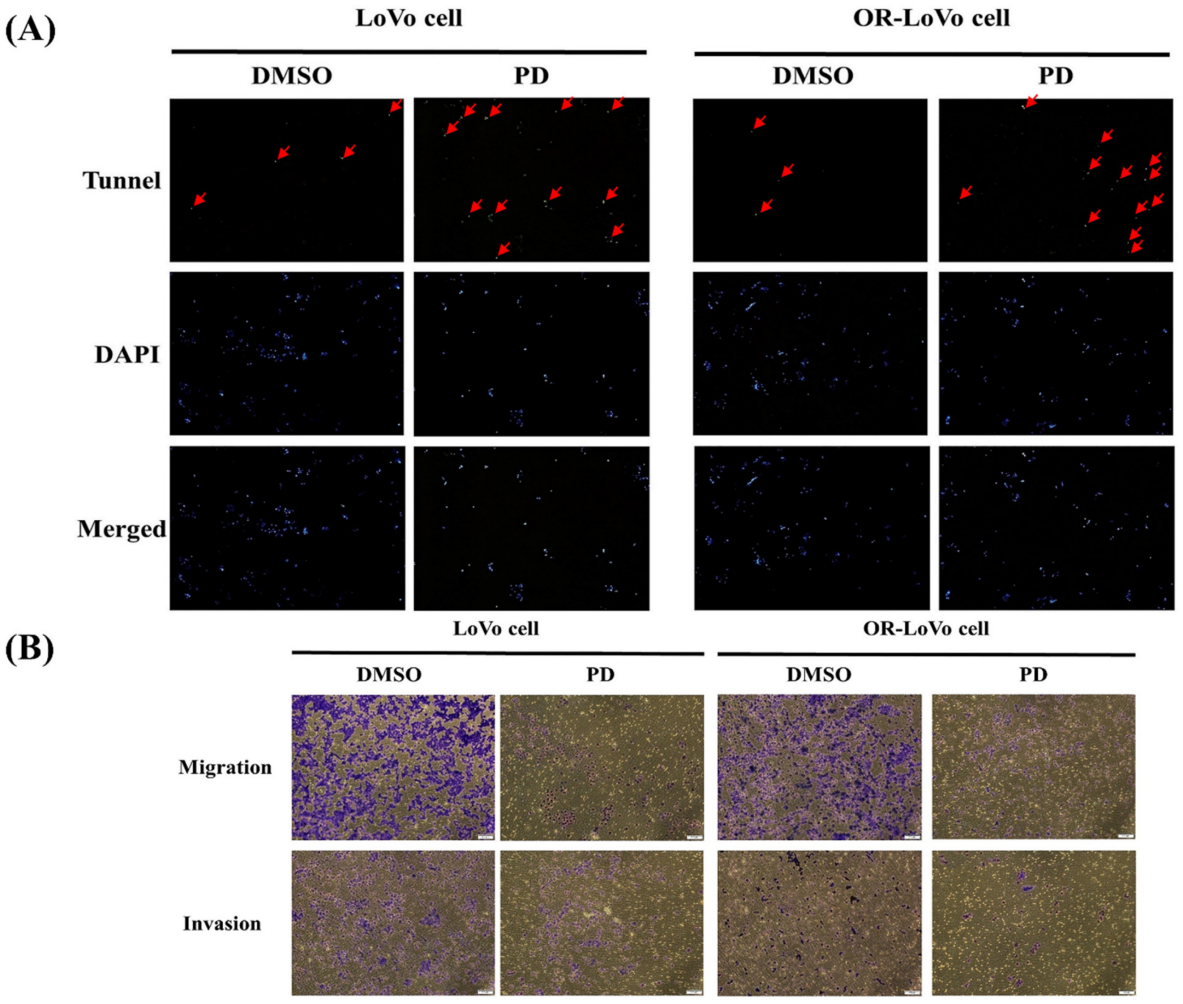
PD induces apoptosis, invasion, and migration in LoVo and OXP-LoVo cells. (A) TUNEL positive cells were increased in LoVo and OXP-LoVo cells after PD treatment. (B) PD treatment effectively reduced the metastasis in LoVo and OXP-LoVo CRCs by inhibiting invasion, and migration.

The effect of PD on apoptosis and metastasis was analyzed in the CRC cells. The number of TUNEL positive cells was significantly higher in the PD treated parental and OXP-LoVo cells when compared to their respective control groups (Figure 5A). Effect PD on metastasis was estimated by invasion, and migration assay. PD treatment in both type of cells effectively prevents the invasion, and migration of the CRCs (Figure 5B), the anti-metastasis effect of PD.

### PD inhibited Yap nuclear translocation and activation in LoVo and OXP-LoVo cells

**Figure 6:**
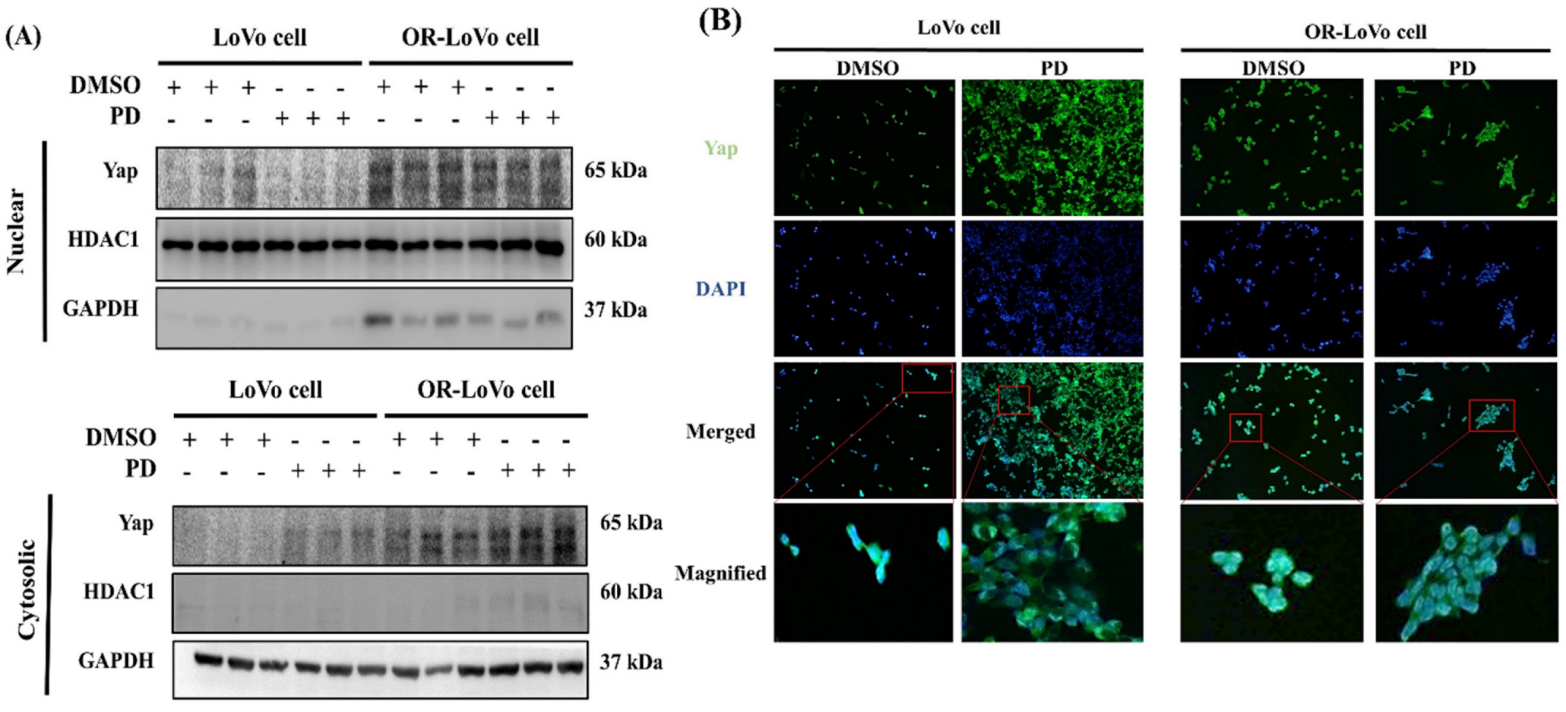
PD inhibited Yap nuclear translocation and activation in LoVo and OXP-LoVo cells. (A) Western blot results showing YAP1 expression in nuclear and cytosolic fractions of PD treated LoVo and OXP-LoVo cells. (B) Immunofluorescence image showing the effect of PD in inhibiting YAP1 nuclear translocation.

Inhibiting YAP activation and nuclear translocation might be an approach to reduce cancer progression. Then we test the effect of PD on YAP activation and nuclear translocation. We quantified the YAP expression in the nuclear and cytoplasmic extract of parental LoVo cells and OXP-LoVo cells. YAP expression in the nuclear extract was increased in OXP-LoVo cells compared to LoVo cells, however, PD treatment reduced the YAP levels in the nuclear extract. In the cytoplasmic extract, PD treatment increased YAP levels in OXP-LoVo cells (Figure 6A). Immunofluorescence assay also confirms that PD treatment in the LoVo cells and OXP-LoVo cells effectively prevents the nuclear translocation of Yap (Figure 6B).

### PD activates and promotes YAP1 degradation through the ubiquitination and proteasome pathway

**Figure 7:**
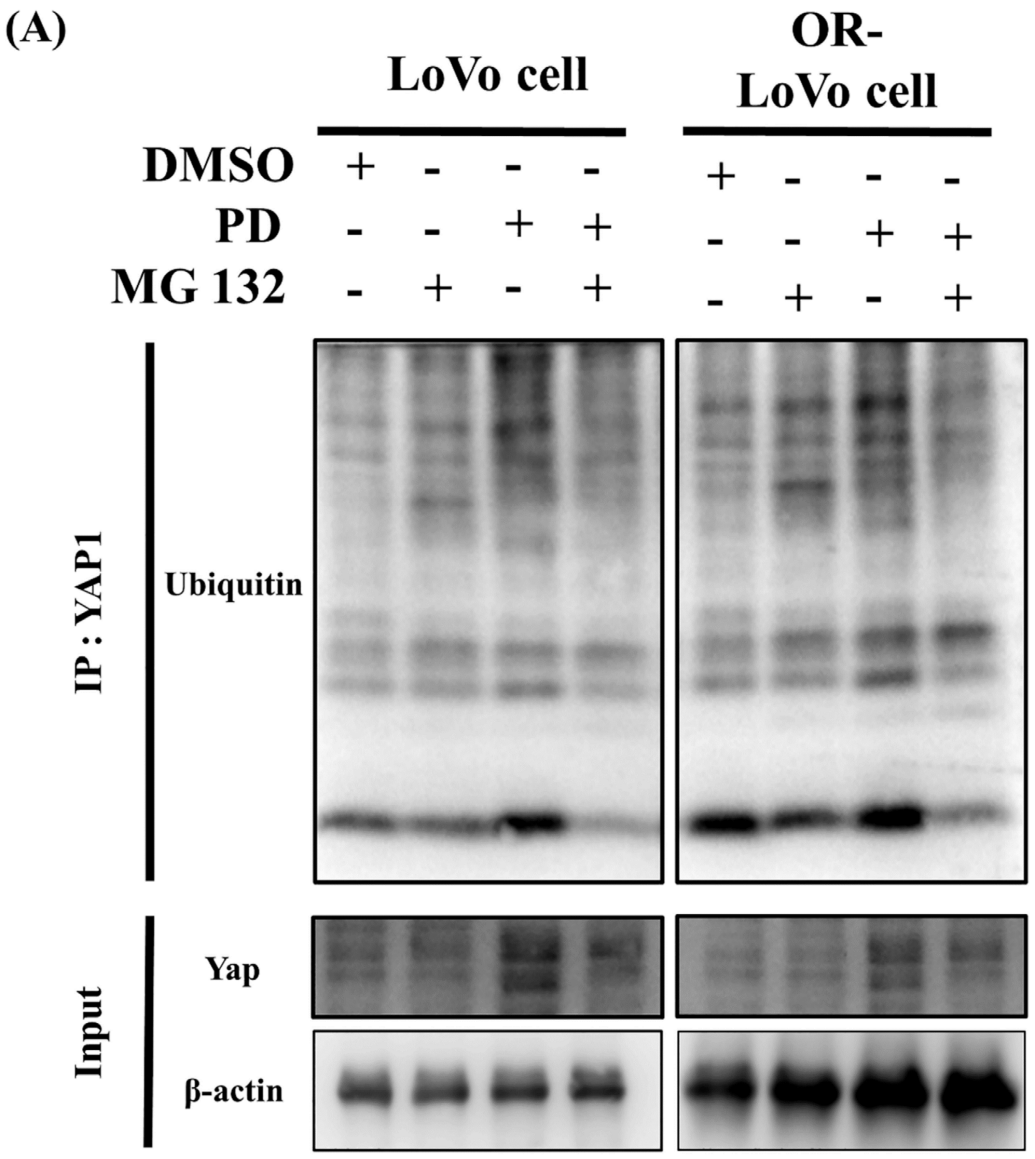
PD activates and promotes YAP1 degradation through the ubiquitination and proteasome pathway.

Immunoprecipitation assay followed by immunoblot was performed for the YAP1 to study the effect of PD on ubiquitination and proteasome pathway. MG132, a proteasome inhibitor was used. PD treatment in LoVo cells and OXP-LoVo cells degrade YAP1 through activating ubiquitination mediated proteasome degradation (Figure 7).

## Discussion

PD has been reported to have an anti-cancer effect in different *in vitro* and *in vivo* 31. PD has been reported to reduce cell viability and invasion while greatly increasing apoptosis in OSCC cells in a dose-dependent manner 33. Further, PD demonstrates the anti-cancer properties in breast cancer cells *in vitro* and *in vivo*, as evidenced by decreasing cell proliferation and survival of MDA-MB-231 cells and inhibited the tumor cell volume in MDA-MB-231 xenograft tumors in BALB/c nude mice 34. In liver cancer cells, PD exerts an anti-cancer effect by inducing apoptosis and inhibiting metastasis 35. PD increased the expression of Fas and FasL at the mRNA and protein levels in HaCaT cells, causing proteolytic cleavage of caspase-8 and -3 in a time-dependent manner, and elicits extrinsic apoptosis 36. In HepG2 and MCF-7 cancer cells, PD dramatically decreased the Bcl-2/Bax ratio and elevated the expression of cleaved caspase-9, caspase-3, and Poly (ADP-ribose) polymerase (PARP) 35, 37. Furthermore, it has been discovered that platycodin D could cause G2/M phase arrest in PC3 cells 38. It has been demonstrated that platycodin D inhibits the migration and invasion of MDA-MB-231 cells in a dose-dependent manner. While MMP-2 activity was only marginally lowered, PD significantly suppressed MMP-9 activity. MMP-9 mRNA expression shown to be down-regulated by PD 39.

Dysfunction of the hippo pathway has been linked to the development and metastasis of CRC 40, 41. Upregulation of hippo pathway proteins such as YAP and TAZ along with other proteins Transcriptional enhancer factor TEF-1 also known as TEA domain family member 1 (TEAD), and Octamer-binding transcription factor 4 (OCT4) have a correlation of developing adenoma to CRC 42. YAP and TAZ upregulations were shown to be substantially linked with lymph node metastases in CRC patients 43. TAZ was sued as a prognostic marker for CRC which is also linked to downstream target genes AXL and Connective tissue growth factor (CTGF) 44.

Extracellular matrix (ECM), cell adhesion, serine/threonine kinase receptor, G protein-coupled receptor (GPCR), and cellular metabolism dysfunction are the direct upstream regulators of YAP/TAZ activity 45. In a clinical study, YAP expression in a CRC patient have shown to regulate epithelial-mesenchymal transition (EMT) by activating Slug and inhibiting E-cadherin 46. Higher YAP expression is linked to CRC recurrence in human CRC hepatic metastases 40. Recently, we have shown that Irinotecan (CPT-11) resistant CRC cells have shown to increase the expression GPCR such as Gαi‐2, Gαq/11, and Gαs and metastasis 47. Nuclear translocation of YAP and upregulation of β-catenin expression could decrease the overall and progression-free survival in CRC patients 43. Another study correlates the YAP/TAZ expression with the chemoresistance CRC with liver metastases and suppressing/inhibiting YAP/TAZ expression in the CRC patients sensitize the CRC to the chemotherapeutic drug cetuximab and increase progression-free survival 48. In our present study, PD treatment dose-dependently decreased LATS2/YAP1 hippo signaling and survival marker p-AKT expression, as well as increased cyclin-dependent kinase inhibitor proteins such as p21 and p27 expression.

Hippo signaling is a piece of epigenetic machinery that regulates the development and progression of cancer. According to the recent *in silico* analysis, promotor sites of the YAP/TAZ genes are greatly enhanced with the binding sites of many transcriptional factors including P300 which acts as a histone acetyltransferase (HAT) at the YAP1 promoter and procured chromatin remodelers of other DNA or histone-modifying components such as CTCF and HAT 16.

In recent years, hippo signaling in many human cancer types has been studied which provides a better understanding of their role in cancer progression. Targeting the YAP/TAZ transcriptional network chemical inhibitors might be a significant step forward in the combat against cancer therapy resistance 49. The nuclear-cytoplasmic shuttling of YAP/TAZ has been given considerable attention for its regulatory function 50. YAP/TAZ must be translocated into the nucleus to act as a transcriptional activation factor. Thus, preventing/inhibiting YAP/TAZ activation and nuclear translocation might be an approach to reduce cancer progression. In our present study, we have shown that YAP levels in the nucleus were increased OXP-LoVo cells, whereas PD significantly reduced the nuclear translocation of YAP suggesting PD sensitize OXP-LoVo cells by inhibiting nuclear translocation of YAP.

A combinatorial treatment regime is the main element of chemotherapy-resistant cancer treatment that focus to sensitize chemotherapy-resistant cancer through the sequential regime of an adjuvant drug 51. Results of our present study demonstrate that combined treatment of oxaliplatin and PD reduce the cell viability significantly in OXA-LoVo cells in a dose-dependent manner. PD dose-dependently decreased LATS2/YAP1 hippo signaling and survival marker p-AKT expression, as well as increased cyclin-dependent kinase inhibitor proteins such as p21 and p27 expression. Importantly, PD activates and promotes YAP1 degradation through the ubiquitination and proteasome pathway. The nuclear transactivation of YAP was significantly reduced under PD treatment, leading to transcriptional inhibition of the downstream gene, including proliferation gene, pro-survival gene, and EMT-related gene. In conclusion, our results showed that PD is suitable as a promising agent for overcoming oxaliplatin-resistant colorectal cancer.

## Figures and Tables

**Figure 8:**
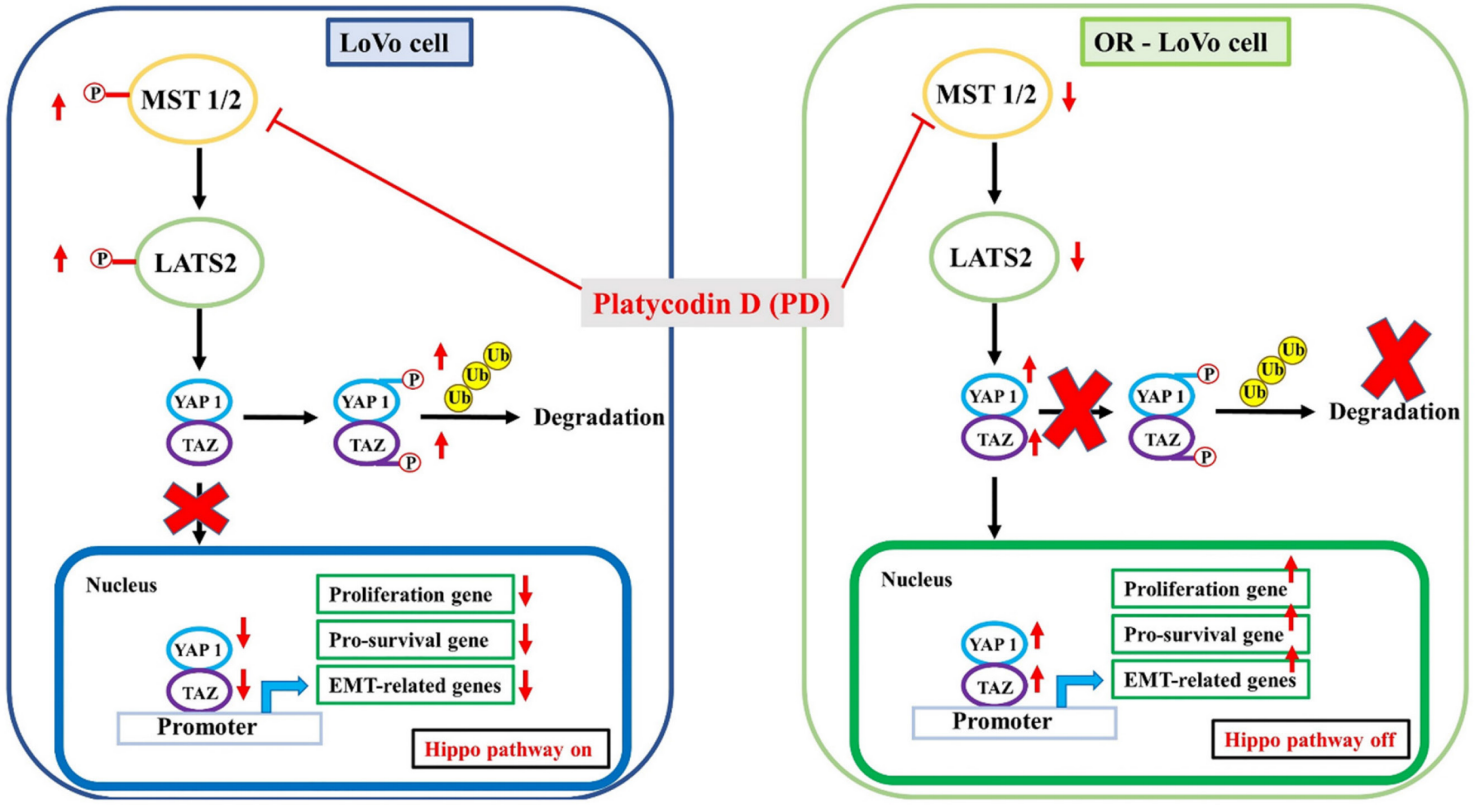
Schematic representation of the molecular mechanism of PD in inhibiting hippo pathway in parental and OXA-resistance LoVo colorectal cancer cells.
